# Dealing with Posttraumatic Nightmares

**DOI:** 10.1192/j.eurpsy.2022.1735

**Published:** 2022-09-01

**Authors:** C. Alvarez Garcia, L. Nocete Navarro, A. Sanz Giancola

**Affiliations:** Hospital Universitario Príncipe de Asturias, Psychiatry, Alcalá de Henares, Spain

**Keywords:** nightmares, posttraumatic, Treatment, PTSD

## Abstract

**Introduction:**

Posttraumatic nightmares are one of the most frequent symptoms in posttraumatic stress disorder. Prevalence can be up to 96%. These nightmares evoke the experienced traumatic event, causing a negative impact. Besides, they are and independent risk for suicide. There are different pharmacological and non-pharmacological options for PTN, despite is no optimal treatment.

**Objectives:**

To analyse the different treatment options for PTN.

**Methods:**

This was a narrative literature review.

**Results:**

The two main treatments for PTN nowadays are the Imagery Rehearsal Therapy (IRT) and prazosin. IRT is a cognitive-behavioral intervention, that helps the patient to change the content of the nightmare to a “happier ending”. Prazosin is an alpha-adrenergic receptor antagonist that blocks the stress response in the central nervous system receptors. Although it was a promising drug, significant differences compared to placebo have not been found. There is growing data that suggests nabilone, a synthetic cannabinoid, could be helpful in PTN treatment. A clinical trial made in Canada revealed that 72% of patients experienced a complete disappearance or at least an important reduction of PTN.

**Conclusions:**

PTN is a very common and distressing symptom in patients presenting PTSD. Nevertheless, there is no treatment with enough evidence for this pathology. On this account, it is fundamental to do more research in order to find and suitable treatment that can improve the quality of life of these patients.

**Disclosure:**

No significant relationships.

